# Targeted Temperature Management and Multimodality Monitoring of Comatose Patients After Cardiac Arrest

**DOI:** 10.3389/fneur.2018.00768

**Published:** 2018-09-11

**Authors:** Peggy L. Nguyen, Laith Alreshaid, Roy A. Poblete, Geoffrey Konye, Jonathan Marehbian, Gene Sung

**Affiliations:** Department of Neurology, Keck School of Medicine, University of Southern California, Los Angeles, CA, United States

**Keywords:** cardiac arrest, targeted temperature management, anoxic brain injury, EEG, prognosis, multimodality monitoring

## Abstract

Out-of-hospital cardiac arrest (CA) remains a leading cause of sudden morbidity and mortality; however, outcomes have continued to improve in the era of targeted temperature management (TTM). In this review, we highlight the clinical use of TTM, and provide an updated summary of multimodality monitoring possible in a modern ICU. TTM is neuroprotective for survivors of CA by inhibiting multiple pathophysiologic processes caused by anoxic brain injury, with a final common pathway of neuronal death. Current guidelines recommend the use of TTM for out-of-hospital CA survivors who present with a shockable rhythm. Further studies are being completed to determine the optimal timing, depth and duration of hypothermia to optimize patient outcomes. Although a multidisciplinary approach is necessary in the CA population, neurologists and neurointensivists are central in selecting TTM candidates and guiding patient care and prognostic evaluation. Established prognostic tools include clinal exam, SSEP, EEG and MR imaging, while functional MRI and invasive monitoring is not validated to improve outcomes in CA or aid in prognosis. We recommend that an evidence-based TTM and prognostication algorithm be locally implemented, based on each institution's resources and limitations. Given the high incidence of CA and difficulty in predicting outcomes, further study is urgently needed to determine the utility of more recent multimodality devices and studies.

## Introduction

Out-of-hospital cardiac arrest (CA) remains a leading cause of sudden morbidity and mortality. Based on recently published statistics from the American Heart Association, 1 of every 7.4 people in the United States will die of sudden cardiac death; however, in those who survive, outcomes have continued to improve over time (1). Enhanced recovery in CA-survivors is likely a product of multifactorial system-based changes, including the advent and evolution of targeted temperature management (TTM).

A multidisciplinary approach is necessary in the care of this complex and critically-ill population. Patients are managed by a team of physicians, nurses and ancillary staff. Neurologists and neurointensivists are often instrumental in the selection of TTM candidates and are central to patient care and prognostication. Early consultation with neurologists might increase the use of therapeutic hypothermia (TH) (2). Despite advances in the medical management of out-of-hospital CA, predicting outcomes remains a challenging task. Established prognostic tools have needed to be re-visited with the advent of TTM (3), and the literature has been limited by heterogenous definitions, non-standardized study methods, small sample sizes, and publication bias. We therefore completed this review to highlight the clinical use of TTM, and to provide an updated summary of multi-modality monitoring and prognostication following CA in a modern neuroscience intensive care unit (ICU). Given the neuroprotective effect of TTM and its impact on prognostication, being familiar with current guidelines and literature is crucial to optimizing patient care.

## Pathophysiology of anoxic injury following cardiac arrest

Hypoxic-ischemic encephalopathy remains a primarily clinical diagnosis; however, the molecular pathophysiology has been studied in both animal and human models. Post-cardiac arrest syndrome, a term used to describe the resuscitative phase that follows return of spontaneous circulation (ROSC), encompasses four key processes: post-CA brain injury, post-CA myocardial dysfunction, systemic ischemia and reperfusion injury and a persistent precipitating pathology (4). Brain injury in the post-CA period is thought to be mediated by multiple pathways that result in ischemic injury, impaired cerebral autoregulation, and the development of diffuse cerebral edema (4).

During CA, the brain is exquisitely sensitive to global ischemia. Neuronal injury occurs when the severity and duration of ischemia is sufficient to cause depolarization of the neuronal plasma membrane. In animal models, this has been demonstrated to occur when blood flow falls below 10 mL/100 g per minute (5, 6). Animal studies have also demonstrated that loss of consciousness is experienced as early as 10 s following circulatory arrest, while an electroencephalogram (EEG) demonstrates isoelectric activity as early as 20 s after the event (7). When ischemic depolarization is brief with subsequent reperfusion, permanent glial injury may not occur; however, when the depolarization is more prolonged, a cascade of metabolic changes mediated by alterations in adenosine triphosphate (ATP), neurotransmitter pathways, mitochondrial dysfunction and calcium homeostasis occurs, which may lead to irreversible neuronal death. Ischemic depolarization >30 min most likely results in neuronal cell death regardless of reperfusion (5).

The exact mechanisms underlying ischemic injury following CA are poorly understood but are likely to involve multiple signaling pathways leading to disruption of cellular homeostasis. At a molecular level, global ischemia causes a depletion of intracellular ATP, with subsequent failure of ATP-dependent ionic channels. This results in accumulation of interstitial potassium with subsequent cell membrane depolarization. The eventual influx of sodium, chloride, and water into cells forms the basis of cytotoxic edema, which occurs during CA and other neurologic injuries. In addition, membrane depolarization results in the intracellular accumulation of calcium with activation of voltage gated calcium channels, as well as release of excitatory amino acid transmitters such as glutamate, perpetuating calcium and sodium influx into the neuron (5, 8).

If global ischemia occurs without subsequent reperfusion, failure of ATP production will lead to a final common pathway of cellular necrosis. Disruption of calcium homeostasis is thought to be one of the primary mechanisms of neuronal cell injury (9). Accumulation of calcium ions in the mitochondria increases free radical production and mitochondrial permeability transition (MPT), resulting in mitochondrial swelling, oxidative damage, loss of ATP production, and cell death. Cellular accumulation of calcium in the cytosol additionally triggers lipolysis, membrane destabilization, proteolysis, nitric oxide production, and endonuclease DNA degradation (5, 8). *In vivo* measurements of calcium demonstrate that in the acute ischemic period, cytosolic levels increase exponentially within 8 min of ischemia in vulnerable brain regions, although levels can return to baseline within 30–60 min if reperfusion occurs (5, 10).

In the setting of ROSC with cerebral reperfusion, additional ischemic injury can still occur. In particularly vulnerable neuronal subpopulations, including in the cortex, hippocampus, cerebellum, corpus striatum, and thalamus, degeneration can occur over hours to days (4). Secondary neuronal death is also mediated by disruption in calcium homeostasis, as levels of cytosolic calcium have been shown to increase up to 24 h after the primary insult in susceptible brain regions (5). Additional inflammatory processes involving cytokines are implicated in peripheral tissue ischemia. Cytokines such as interleukin-6 (IL-6) and tumor necrosis factors (TNF) perpetuate delayed ischemia in the reperfusion period. Animal models have demonstrated that rapid infiltration of neutrophil and pro-inflammatory T-lymphocytes into the brain occur as early as 3 h following ROSC, and last up to 3 days (11).

## A paradigm shift: targeted temperature management in the modern ICU

For the last two decades, TTM, previously referred to as therapeutic hypothermia, has been a growing research topic in the ICU literature. Currently, given its neuroprotective role after CA with improvement of neurologic outcomes, TTM is considered the standard of care for survivors of CA who present with coma after ROSC (12).

### Mechanisms of neuroprotection with hypothermia

The manner that hypothermia acts as a neuroprotectant is still not fully understood. In animal models, hypothermia has been shown to limit several microscopic events, including the inhibition of inflammatory cytokine release (ex. interleukins, prostaglandins), that normally lead to apoptosis and cell death (13–15). At a tissue level, hypothermia was found to reduce cerebral metabolic demand (approximately 6–8% per 1°C), cerebral edema, and intracranial pressure (ICP), while also increasing seizure threshold (16).

### Targeted temperature management

TTM was first introduced in the 1950s and 1960s, primarily in animal models. Many of these early studies showed that both moderate systemic hypothermia (32–30°C) (17) and mild hypothermia (34°C) (18–20) cause decreased brain damage after CA in animals. The subject of TH somewhat faded until it was reintroduced in human subjects in the early 2000s by two important randomized trials (RCTs): a study by Bernard et al, and the Hypothermia after Cardiac Arrest Study Group (21, 22). These studies were the first to provide Class I evidence supporting the use of TTM for CA survivors to improve both survival and functional outcomes using mild hypothermia (32°-34°C for 12 or 24 h). Based on these findings, the International Liaison Committee on Resuscitation (ILCOR) recommended in 2003 that CA-survivors in coma should receive TH when the initial rhythm was ventricular fibrillation, with potential benefit with other rhythms (23).

These recommendations have since been under continuous review and change. Three major clinical trials and a meta-analysis by Kim et al failed to show definite improvement in outcomes in patients with non-shockable rhythm (24–27). Similarly, TTM does not appear to benefit patients who sustain in-hospital CA in terms of mortality or neurologic recovery (28). Regarding the timing and duration of TTM and depth of TH, best practices are unknown. Pre-hospital initiation of TTM has not been shown to improve outcomes (29, 30). Prolonged cooling also appears to have no important clinical effect. Kirkegaard et al compared 24 vs. 48 h of cooling to 33°C and found no significant differences in neurologic outcomes (31). While early guidelines recommend mild hypothermia, a recent RCT found no change in neurologic recovery or survival at 6 months when comparing 33° and 36°C (32).

Current guidelines do not recommend a specific cooling method for TTM; however, multiple methods are available for induction and maintenance, including both invasive and non-invasive devices (33, 34). Recent data suggest that endovascular cooling is more rapid and may be more effective in maintaining TTM, although surface cooling may be as effective during induction (35, 36); however, clinical trials to-date have not shown effect on overall mortality and neuro-sequelae between invasive and non-invasive methods. Alternative devices, including trans-nasal, peritoneal and esophageal cooling are under investigation, but no recommendations for their use can be made currently (37–39).

The current American Heart Association, Canadian TTM guidelines, and American Academy of Neurology practice parameters are considered the most evidence-based clinical recommendations and the standard of care for managing post-CA patients who remain in coma after ROSC (12, 40, 41) (Table 1). Further research is needed to study different aspects of these guidelines to achieve the best clinical results.

**Table 1:** Summary of recent guidelines on targeted temperature management for out-of-hospital cardiac arrest patients.

**Committee**	**Summary of recommendations**
2015 American Heart Association (12)	Induce hypothermia for unconscious adult patients with ROSC after OHCA when the initial rhythm was VF or pVT (class I, level of evidence: B-R) Similar therapy may be beneficial for patients with non-VF/non-pVT (non-shockable) OHCA or with in-hospital arrest (class I, level of evidence: C-EO) The temperature should be maintained between 32°-36°C (class I, level of evidence: B-R) It is reasonable to maintain TTM for at least 24 h (class IIa, level of evidence: C-EO) Routine prehospital cooling of patients with ROSC with IV rapid infusion is not advised (class III: no benefit; level of evidence A) It is reasonable to prevent fever in comatose patients after TTM (class IIb, level of evidence C-LD) Hemodynamically stable patients with spontaneous mild hypothermia (>33°C) after resuscitation from cardiac arrest should not be actively rewarmed
2016 Joint Statement from The Canadian Association of Emergency Physicians, the Canadian Critical Care Society, Canadian Neurocritical Care Society, and the Canadian Critical Care Trials Group (40)	We recommend that patients who suffer out-of-hospital cardiac arrest are eligible for TTM (High quality, strong recommendation) We recommend that TTM can be initiated in any medical environment with the necessary supports, including prehospital, ED and critical care unit (Moderate quality, strong recommendation) We recommend that clinicians attempt to achieve target temperature as rapidly as possible (Low quality but strong recommendation) We do not recommend a specific cooling method for TTM.” We recommend that patients undergoing TTM should receive sedation and analgesia We suggest that paralytics be used during induction and rewarming phases of TTM, to facilitate tight temperature control and to prevent shivering
2017 American Academy of Neurology Practice Guidelines (41)	Comatose patients after CA in whom the initial cardiac rhythm is VT or VF, TH is likely effective in improving neurologic outcome and survival (Level A) Comatose patients after CA in whom the initial cardiac rhythm is VT/VF or PEA/asystole should not be offered prehospital cooling with 2 liters 4°C IV fluid or intranasal cooling (Level A) Comatose patients after CA in whom the initial cardiac rhythm is either VT/VF or PEA/asystole, TTM (33°C for 24 h followed by 8 h of rewarming to 37°C and maintained below 37.5°C until 72 h) is likely as effective as TH in improving neurologic outcome and survival and is an acceptable alternative to TH (Level B) In comatose patients after CA, the addition of coenzyme Q10 to TH possibly improves survival but does not improve neurologic status at 3 months and may be offered (Level C) No recommendations are made on the following (Level U): TH when the initial cardiac rhythm is PEA/asystole Use of 32° vs. 34°C TH Use of invasive cooling instead of surface cooling Use of standardized protocols for TH Use of epoeitin alfa in addition to mild TH

*ROSC, return of spontaneous circulation; OHCA, out-of-hospital cardiac arrest; VF, ventricular fibrillation; pVT, pulseless ventricular tachycardia; IV, intravenous; TTM, targeted temperature management; R, randomized; EO, expert opinion; LD, limited data; VT, ventricular tachycardia; TH, therapeutic hypothermia; PEA, pulseless electrical activity; IV, intravenous*.

## Neurophysiologic testing after cardiac arrest

### Use of EEG studies

Beyond the clinical exam, EEG is one of the most widely used prognostic tools after anoxic brain injury from CA. Areas most susceptible to hypoxic-ischemic injury, including the cerebral cortex and hippocampus (42) are highly epileptogenic and are feasibly captured by surface EEG. From a practical aspect, it is an attractive study because it is non-invasive, can be performed rapidly, and is available at most hospitals managing CA patients. Clinical seizures are reported from TTM trials to occur in over 25% of patients (32), while malignant EEG features are recorded in up to 86% of patients who remain comatose after resuscitation and re-warming (43). Non-convulsive seizures are thought to occur in approximately one-quarter of comatose patients after cardiac arrest (44, 45).

#### Timing and duration of EEG

The optimal timing for EEG recording after CA is unknown. Since sedation is required for TH and TTM, EEG is often reserved for those who fail to awake after rewarming; however, early monitoring may be indicated. In study populations undergoing TTM, most seizures on continuous EEG (cEEG) occur within 12 h of resuscitation and prior to achieving normothermia (44, 45). Malignant patterns in the first 8 h of cEEG have also demonstrated a high positive predictive value for poor outcome (46). Although this suggests utility in early cEEG, studies have shown that intermittent short-duration EEG has potentially similar diagnostic and prognostic yield, with no difference in clinical outcomes, and is associated with reduced per patient cost (47, 48). Given the lack of evidence to suggest treatment of seizures and malignant EEG findings improves outcomes (49, 50), the principal use of intermittent EEG as a prognostic tool is reasonable in centers where cEEG is not readily available.

#### Prognostic value of EEG findings

Electroencephalogram findings most thought to predict poor neurologic outcome includes absence of EEG reactivity, alpha coma, low-voltage recording, burst-suppression pattern, and status epilepticus (49). Several older and newer grading scales that account for TTM have been proposed to classify EEG findings after CA (50–54); however, none have achieved widespread adoption. This may in part be due to a difficulty of interpreting and understanding the clinical implications of complex EEG findings for non-electroencephalographers. A simplified system might differentiate between “benign” and “malignant” EEG patterns (43, 55).

##### EEG features in patients who do not undergo therapeutic hypothermia

A low-voltage EEG (≤21 μV) reflects neuronal dysfunction and is a reliable predictor of poor outcome in those who do not receive TTM. In two larger studies, low-voltage EEG seen at 24–72 h after resuscitation was associated with poor outcome with a 0% false positive rate (FPR) (56, 57). Similarly, myoclonic status epilepticus (MSE) when differentiated from a benign form of chronic myoclonus, Lance-Adams syndrome, has been invariably associated with poor outcome in those who are not treated with TH (58). Although electrographic seizure other than MSE, alpha coma and burst suppression are considered useful prognostic findings, they are not invariably associated with poor outcome in the CA population (53, 57, 59, 60).

##### EEG features in patients treated with therapeutic hypothermia.

TTM improves neurologic outcomes following CA (32) but lowering intracranial temperature and the sedation necessary for TH confounds EEG interpretation. Its accuracy as a prognostic tool has had to be re-evaluated with the advent of TTM. Although early post-hypoxic myoclonus is associated with poor prognosis, since the publication of the 2006 American Academy of Neurology Guidelines on prognostication following CA (61), several authors have reported outcomes better than predicted in patients with myoclonic seizures (44, 50, 62–65). Cortical myoclonus or status myoclonus within 72 h from CA did not exclude a good neurologic recovery defined as Cerebral Performance Category (CPC) 1 or 2, with a 5% FPR (44, 50, 62–64). Some have suggested that “benign” and “malignant” myoclonus or MSE can be differentiated by the background EEG pattern (65, 66).

Status epilepticus (SE) other than MSE strongly predicts poor outcome (CPC 3–5) (45, 50, 67); however, good outcome in CA patients is possible. In one study of 106 comatose CA survivors, 2/33 SE patients achieved good neurologic outcomes (66). Like MSE, EEG background is an important factor. In a study of cEEG in TTM patients, those with SE arising from burst-suppression invariably had poor neurologic recovery, while good outcomes were possible in those with SE arising from an EEG with continuous background (4% FPR) (68).

A burst-suppression EEG background or low-amplitude recording alone does not predict poor outcome with 100% specificity (64, 68, 69). Similarly, a non-reactive EEG background is compatible with neurologic recovery with a 3% FPR when recorded during TH (50, 62, 67), while in one larger study, three patients with absence of EEG reactivity within 72 h of resuscitation achieved good outcomes (63). Although prognosis is not invariably poor in patients undergoing TTM with a single malignant EEG feature, the presence of multiple features significantly increases the likelihood of poor outcome. In a large cohort of patients enrolled in a TTM trial, a single malignant EEG feature had a low specificity for poor prognosis (48%), which increased to a 96% positive predictive value when two malignant EEG features were present (43).

#### Continuous EEG using depth electrodes

In neurocritical care, EEG depth electrodes are inserted through small burr holes, often in combination with other multimodality brain monitors to capture electrographic activity below the cortex. Their use in the management and prognosis of CA survivors has not been extensively studied in any large trial. In one case report, authors demonstrated the feasibility of using multimodality monitoring, including depth EEG, to detect SE that was associated with reductions in brain tissue oxygen tension and increase in cerebral blood flow and brain temperature (70). Future study is needed if invasive monitoring that includes depth EEG has additional prognostic value in CA.

#### Limitations

Beyond being confounded by hypothermia and sedative medications, EEG for prognostication after CA has other important limitations. The frequency of EEG and availability of cEEG is dependent on institutional resources and varies widely, with cEEG often only available at larger academic centers. Given the lack of evidence suggesting its superiority to intermittent EEG (49, 50), the American Clinical Neurophysiology Society and other committees have not made guidelines to standardize EEG after CA (71, 72).

EEG interpretation remains a specialized skill, with the large proportion of ICU providers unable to independently interpret studies. Recordings and terminology can be confusing to non-epileptologists. Even among experts, interrater agreement on EEG patterns can vary (73), resulting in many authors proposing standardization to classifying EEG in this population (43, 73). Use of automated, quantitative EEG techniques may find future utility in prognostication and is the subject of current study (51, 74, 75).

### Somatosensory evoked potentials

Somatosensory Evoked Potentials (SSEPs) are often used for prognostication in CA survivors who have a poor neurologic exam after resuscitation and normothermia (76). In the most widely used test, small electrical stimulation (<50 μV) is applied to the median nerve in the arm, followed by recording of post-stimuli waveforms over the primary somatosensory cortex by surface electrodes (76). The bilateral absence of the short-latency N20 waveform is highly specific for poor outcome after CA. In those who are not treated with TH, bilateral absence of the N20 response within the first 7 days showed a 0% FPR (95% CI 0–12] with only 1/432 patients achieving a good outcome (58). In TTM patients, this finding also showed high predictive value for poor outcome both during TH (64, 77, 78) and after rewarming (62, 64, 67, 77, 78), with exception of one case where reappearance of the N20 response corresponded with full neurologic recovery (79). Given these robust findings, early use of short-latency N20 SSEPs for prognostication after CA is endorsed by multiple expert committees (64, 72). The use of longer latency waveforms, such as the N70 response (80), requires further study.

#### Limitations

The optimal timing for SSEP is unknown. Given both peripheral and central conduction times are prolonged during hypothermia (81), most practitioners delay SSEP testing until after the rewarming phase. Using a dichotomous measure of absent vs. present SSEP also significantly limits its sensitivity in predicting poor outcomes. Determining prognostically useful N20 amplitude thresholds should be further studied in future cohorts (82). Importantly, SSEPs are not routinely performed at all hospitals that treat CA survivors, further limiting its adoption in standardized prognostication algorithms.

## Imaging modalities after cardiac arrest

### CT imaging

Brain CT is typically the first neuroimaging study obtained in out-of-hospital CA survivors, as it can be performed quickly and identifies patients who may not benefit from TTM. The primary finding in moderate to severe hypoxic-ischemic injury is cerebral edema characterized by attenuation of affected gray and white matter (83). In a study of TTM trial patients, generalized edema was seen in 9.6% of CT images within 24 h of ROSC and predicted poor outcome (CPC 3–5) with 97.6% specificity (95% CI 91.8–9.4) but only 14.4% sensitivity (95% CI 9.4–21.4) (84). Between days 1 and 7, generalized cerebral edema predicted poor outcome with a 0% FPR. In this study, other CT findings were infarct in 18.9% and intracranial hemorrhage in 6.3% (84).

### The role of standard MRI

Despite MRI being more sensitive for detecting neuronal injury compared to CT, because of longer acquisition times and more necessary resources, its early use during the pre-TH and TH phase is limited. The optimal time window for MRI imaging is unknown; however, prognostic accuracy may be greatest between 2 and 5 days, with reduced sensitivity if done within 24 h (85, 86).

Brain areas most susceptible to hypoxic-anoxic injury include the cerebral cortex and deep brain nuclei that include the caudate, basal ganglia and thalamus. Diffusion restriction involving these areas, characterized by hyperintensity on diffusion-weighted imaging (DWI) and hypointensity on apparent diffusion coefficient (ADC) images, predicts poor outcome with high specificity (85, 87, 88). In a study correlating MRI findings with neuron-specific enolase, a biomarker with high prognostic value, levels >33 μg/ml after CA was associated with extensive DWI changes in both deep nuclei and the cortex (89). These patients had invariably poor outcome. More recently, quantitative measures of diffusion restriction burden on MRI has been proposed to standardize post-CA prognostication (86, 87, 90, 91); however, the most appropriate thresholds have not been established. In one multi-centered study, an ADC value <650 × 10^−6^m^2^/s in ≥10% of the total brain volume independently predicted poor outcome with a specificity of 91% (95% CI 75–98) and sensitivity of 72% (95% CI 61–80), with >22% of brain volume needed to achieve 100% specificity (90). In another study, <15% total brain volume with significant diffusion restriction was predictive of good neurologic recovery (91). As part of multimodality monitoring, combining MRI findings with EEG features, biomarkers and clinical exam may result in the highest accuracy in predicting outcomes (89, 91). Currently, the implementation of MRI in post-CA prognostication algorithms has not been strongly recommended by consensus guidelines (72).

### Other imaging modalities

#### Functional imaging

There are a limited number of studies with small sample sizes that have investigated the use of functional MR imaging (fMRI) in the management and prognosis of CA survivors (92). In a positron emission tomography (PET) pilot study of 7 post-CA patients, all initially demonstrated a low cerebral metabolic rate of oxygen metabolism (CMR02) and cerebral blood flow; however, at day 7, those with persistent coma had lower CMR02 compared to those who regained consciousness (93). More recently, fMRI connectome imaging has demonstrated potential as a prognostic tool in CA, where patients who achieved favorable outcome have higher default-mode network connectivity (94, 95). It is unclear if fMRI is more accurate in predicting outcome in comparison to other MRI modalities (94), and they have not been compared directly with more established prognostic tools (92). Validation of PET imaging and other fMRI techniques may be a field of future research.

#### Transcranial doppler ultrasound

Transcranial Doppler ultrasound (TCD) is a useful bedside, non-invasive tool for monitoring characteristics in cerebral blood flow and intracranial vascular resistance. Early TCD studies suggested that higher vascular resistance indices and post-resuscitation hyperemia predicted poor outcome (96, 97); however, subsequent larger studies have not found correlation between TCD measurements and outcomes during both the TH phase and after rewarming (98, 99). TCDs in CA survivors is not part of routine practice in most hospital centers.

## Other multimodality monitoring of comatose patients after cardiac arrest

### Use of invasive and noninvasive multimodality monitors

Following CA, global anoxia leads to derangements of cerebral oxygenation and metabolism. Multimodality monitoring, including invasive ICP monitors, microdialysis catheters, jugular bulb catheters, near-infrared spectroscopy (NIRS) and monitors of brain oxygen tension, discussed here, aim to provide the clinician with physiologic parameters that reflect cerebral perfusion, tissue oxygenation and degree of metabolic derangement to help guide treatment and minimize secondary brain injury. Its use in other forms of neurologic injury, including traumatic brain injury and subarachnoid hemorrhage, have been studied extensively, but its use in survivors of CA remains poorly established in clinical practice.

### Brain oxygen tension monitors

Direct measurement of brain oxygen tension (PtiO2) or brain tissue oxygenation (PbtO2) is performed using a catheter similar in size to an ICP monitor, often through a burr hole or craniotomy site. Currently, it is primarily utilized in traumatic brain injury (TBI) populations, where studies have demonstrated its use may help guide treatment; however, the evidence for PbtO2 monitoring improving outcomes is inconsistent and its recommendation was removed from the most recent Brain Trauma Foundation Guidelines (100). PbtO2 monitors have the disadvantage of being limited to measuring focal ischemia, which may not be adequate in CA patients, and requiring daily calibration (101).

In animal models, mostly porcine, brain tissue oxygenation has been measured during CA and cardiopulmonary resuscitation (CPR). In one model, PbtO2 decreased to near zero during arrest, but increased with initiation of CPR and, if ROSC was achieved, exceeded pre-arrest values by 4-fold (102). Similar findings were seen in another animal model, although PbtO2 did not increase during CPR and only recovered with achievement of ROSC (103). In a recent porcine model of CA, hemodynamic-directed CPR was compared to depth-targeted CPR. In the study, a higher PbtO2 was achieved with hemodynamic-directed care that utilized vasopressors to maintain a target coronary perfusion pressure >20 mmHg (104). In humans, one case report demonstrated that feasibility of PbtO2 monitoring during CPR in an adult patient who achieved ROSC (105). Theoretically, PbtO2 monitoring would provide accurate and real-time measurements of brain oxygenation to ensure cerebral perfusion is maintained during CPR, ROSC, and TTM. Larger case series and trials are needed.

### Near-infrared spectroscopy

NIRS is a non-invasive method of monitoring regional oxygenation in the cerebral cortex (rSO_2_). Near-infrared light emitted by one surface probe passing through the cortex is absorbed by biological molecules including oxyhemoglobin, deoxyhemoglobin, and less commonly cytochrome-c oxidase (CCO), at different wavelengths. Differences in absorption by the receiving probe are used to quantifythe proportion of oxygenated hemoglobin or other molecule, which is reflective of perfusion (101). Advantages of NIRS are that it is non-invasive and can be performed at bedside.

Higher values of rSO_2_ on NIRS have been demonstrated in patients achieving ROSC compared to those who do not achieve ROSC (106). Studies have also demonstrated higher values of rSO_2_ using NIRS in post-CA patients with good neurologic outcome, although specific thresholds or cut-offs have not been established. In one study, post-CA patients with good neurologic outcome had higher rSO_2_ values compared to those who with poor outcome (55.6 ± 20.8% vs. 19.7 ± 11.0%, *p* < 0.001, respectively), with a threshold rSO_2_ value of >42% for predicting good outcome yielding a sensitivity of 0.79, specificity of 0.95, PPV 0.41 and NPV 0.99 (107). In comparison, another prospective study found that although post-CA patients with a good outcome had significantly higher median rSO_2_ levels compared to those who had poor outcome (median rSO_2_ of 68% vs. median rSO_2_ of 58%, respectively) there was significant overlap in the range of rSO_2_ between the two groups, limiting its use as a prognostic tool (108).

### Microdialysis catheters

Microdialysis catheters allow frequent assessments of cerebral metabolism and ischemic injury by utilizing a thin dialysis probe that is inserted into brain interstitial tissue. The catheter can measure extracellular solutes that diffuse through the membrane of the probe. Measurable solutes include markers of cerebral metabolism such as CSF lactate, pyruvate, glucose, and markers of excitotoxicity such as glutamate, and cellular damage such as glycerol (109, 110). Additional values of importance, including markers of metabolic crisis such as lactate:pyruvate ratio (LPR), can be calculated autonomously.

During CA, the brain becomes oligemic, leading to diffuse ischemia. Patterns of ischemia are characterized by a decrease in cerebral glucose and an increase in cerebral lactate, which may reflect a state of anaerobic metabolism. These findings, along with increased glutamate and LPR have been demonstrated in animal models of CA (111, 112). Microdialysis results may have additional prognostic value. In rats, survivors of CA after CPR showed significantly higher cerebral lactate and glucose concentrations within 8 min compared to non-survivors, as well as increased lactate, glucose and pyruvate beyond 8 min (111). One small patient series of 4 human subjects with microdialysis catheters who underwent TTM demonstrated elevated LPRs that persisted despite achieving a neurologically intact exam (113). Although glutamate was initially high, values normalized in all 4 patients. Another patient series of CA survivors who underwent TTM found that LPRs progressively increased in those with poor outcomes with significantly higher values on days 2–3 compared to patients with favorable outcomes (114).

These preliminary studies establish the feasibility of microdialysis catheter monitoring in CA patients undergoing TTM. Although it has been demonstrated that neurochemical markers of cerebral ischemia are found after CA, further studies are needed to determine if they have a role in predicting outcome.

### Jugular bulb catheters

Indirect measurements of cerebral oxygenation using jugular bulb catheters have been used in brain injured patients to assess the balance of cerebral oxygen supply and consumption, and to estimate cerebral perfusion (101). The catheters are placed in the internal jugular vein and are directed cranially to measure venous oxygen saturation (SjvO2], with a normal range of 55–70%. In CA, SjvO2 is reduced, reflecting decreased oxygen delivery and increased oxygen utilization in a stress state (101). Disadvantages of jugular bulb catheters are that it is an invasive monitor, with risk of carotid puncture, hematoma formation, or venous thrombosis during prolonged monitoring (101).

In a study of 30 comatose post-resuscitation patients, all subjects had an initial SjvO2 that was lower than the measured mixed venous oxygen saturation (SmvO2) within 6 h of CA, but in 12/20 non-survivors, SjvO2 increased significantly to values higher than the SmvO2 (115). The significance of this finding is unclear. Other studies have demonstrated higher SjvO2 in comatose non-survivors of CA, which was thought to reflect a decrease in cerebral oxygen consumption due to loss of functional brain tissue and may be an indicator of poor neurologic outcome (116). Although it involved a small sample size, the study found that the positive predictive value of a difference between SmvO2 and SjO2 of ≤ 0 for predicting irreversible brain damage at 24 h following CA was 93%, while the negative predictive value of SmvO2–SjO2 > 0 was 53%. Another study of 34 patients found that having a SmvO2 > SjvO2 between 24 and 48 h after ROSC had a positive predictive value of 100% and a negative predictive value of 92% for predicting recovery of consciousness (117). Unfortunately, these results have not been reliably duplicated with TTM patients. In the TTM era, one study of 75 patients found no difference in SjvO2 between patients with good and poor outcome at 6 months (118).

### External ventricular drain and ICP catheters

ICP monitoring devices are typically categorized based on their location of placement, often either ventricular or intraparenchymal, with extradural and subdural monitors being less common. Intraventricular catheters are both diagnostic and therapeutic, allowing clinicians to drain cerebrospinal fluid if ICP rises. Intraparenchymal monitors have the advantage of lower infection rates compared to ventricular catheters; however, they are more prone to drift and technical complications that limit its usability (119).

Following CA, cerebral edema occurs as early as the first 24 h, with CT imaging demonstrating diffuse loss of gray-white differentiation in up to 12% of scans performed and global cerebral edema in as many as 6% (120). ICP monitoring is frequently used when cerebral edema is diagnosed in brain injured patients, including those with traumatic brain injury, intracerebral hemorrhage, or subarachnoid hemorrhage. Uncontrolled intracranial hypertension is associated with poor outcomes in the TBI literature, and although its impact in CA patients is not well defined, ICP elevations can result in cerebral herniation and may serve as a trigger for monitoring and augmenting cerebral perfusion pressures (CPP) (8).

Elevations in ICP have been identified in post-CA patients during the resuscitation period, which may be due to delayed hyperemia (97). In one post-resuscitation study of ICP monitoring performed on 84 patients, peak ICP > 25 mmHg was associated with poor outcomes. Additionally, non-survivors consistently had higher ICPs and lower CPP than survivors (121). This association has been noted in more recent studies following the advent of TTM. Increases in ICP have been noted during both TH and the rewarming period, and at least in one study, all cases of ICP > 25 mmHg were associated with mortality (113, 122). Even when CPP did not differ between groups, studies have shown elevated ICPs are associated with poor outcome (114). The evidence suggests that elevated ICP can occur after CA and during rewarming in patients who undergo TTM; although, to date, no large studies have used ICP monitors to guide management of the post-CA patient.

## Discussing prognosis after cardiac arrest

Despite the use of multiple prognostic tools, prediction of outcomes after CA is difficult. Registries such as the International Cardiac Arrest Registry (INTCAR), a prospective multinational registry of treatment and outcomes in post-CA patients who achieve ROSC, have been developed with the intention of furthering research on CA outcomes (123). The ongoing clinical trial, Multimodal Outcome Characterization in Comatose Cardiac Arrest Patients Registry and Tissue Repository (MOCHA), proposes to develop a multimodal prediction model for outcomes in post-CA patients (124). Currently, given the available evidence in the literature, it is reasonable to discuss pertinent clinical exam, EEG, SSEP, biomarker, and CT and MRI findings with family members and surrogate decision makers to help guide their expectations and inform their decisions. Further validation is needed before fMRI and invasive multimodality monitoring devices are used in standardized prognostication algorithms.

The timing of outcome prediction is crucial in CA. The 72 h clinical exam remains the most informative; however, in patients receiving TTM, the earliest time for prognostication using the clinical exam may be 72 h after achieving normothermia and after other confounding factors, such as paralysis and sedation are safely excluded (12, 64). Similarly, the accuracy of other prognostic tools may be affected by TH, with MRI at day 2–5 being reasonable for prognostication (85, 86).

The accuracy and limitations of prognostication should be shared with all family members and decision makers. Caution must be taken to avoid propagation of a self-fulfilling prophecy in withdrawing life sustaining therapy in this patient population. Studies on prognostication post-CA may suffer from bias as patients with poor neurologic prognosticators may receive limited intensive care or may have had recommendations for withdrawal of treatment (125). Often, goals of care established by family members or decision makers do not align with a patient's prognosis (126), where patient age, comorbidities, prior patient wishes, and cultural aspects factor in to a decision. Establishing and implementing a protocol and expected practice, based on each institution's resources and limitations may help clinicians and families navigate this difficult course.

## Conclusion

Neurologists and neurointensivists are central to guiding TTM, ICU care and prognostication in survivors of CA. Given the neuroprotective effects of TTM and their influence on prognostication and multimodality monitoring, providers should be familiar with the most recent guidelines and literature. We recommend that an evidence-based TTM and prognostication algorithm be locally implemented, based on each institution's resources and limitations. Given the high incidence of CA and the difficulty in predicting outcomes, further study is urgently needed to determine the utility of more recent multimodality devices and studies.

## Author contributions

RP, JM, and GS conceived the manuscript topic and outline. PN, LA, RP, and GK were the primary authors of the manuscript with input from all authors. PN, RP, and JM were involved in critical manuscript revisions. RP, JM, and GS directed manuscript preparation. All authors read and approved the submitted version.

### Conflict of interest statement

The authors declare that the research was conducted in the absence of any commercial or financial relationships that could be construed as a potential conflict of interest. The reviewer CD and handling Editor declared their shared affiliation at the time of the review.
