# Corrigendum: Targeted Temperature Management and Multimodality Monitoring of Comatose Patients After Cardiac Arrest

**DOI:** 10.3389/fneur.2018.00877

**Published:** 2018-10-30

**Authors:** Peggy L. Nguyen, Laith Alreshaid, Roy A. Poblete, Geoffrey Konye, Jonathan Marehbian, Gene Sung

**Affiliations:** Department of Neurology, Keck School of Medicine, University of Southern California, Los Angeles, CA, United States

**Keywords:** cardiac arrest, targeted temperature management, anoxic brain injury, EEG, prognosis, multimodality monitoring

In the original article, there was an error. Near infrared spectroscopy (NIRS) is described under the heading Invasive Multimodality Monitoring of Comatose Patients After Cardiac Arrest and the subheading Use of Invasive Multimodality Monitors when it is a non-invasive modality of monitoring. A correction has been made to the heading, which now reads Other Multimodality Monitoring of Comatose Patients After Cardiac Arrest and the subheading which now reads Use of Invasive and Noninvasive Multimodality Monitors. The authors apologize for this error and state that this does not change the scientific conclusions of the article in any way. The original article has been updated.

## Conflict of interest statement

The authors declare that the research was conducted in the absence of any commercial or financial relationships that could be construed as a potential conflict of interest.

